# Not Extremely Plastic: Testing the Limits of Morphological Plasticity in Fungal Mycelia in Response to Soil Grazers

**DOI:** 10.1111/ele.70281

**Published:** 2025-12-03

**Authors:** Carlos A. Aguilar‐Trigueros, Lynne Boddy, Mark D. Fricker

**Affiliations:** ^1^ Department of Biological and Environmental Science University of Jyväskylä Jyväskylä Finland; ^2^ Center Synergy of Systems (Synosys), Center for Interdisciplinary Digital Sciences Technische Universität Dresden (TUD Dresden University of Technology) Dresden Germany; ^3^ Hawkesbury Institute for the Environment Western Sydney University Penrith New South Wales Australia; ^4^ School of Biosciences, Sir Martin Evans Building Cardiff University Cardiff UK; ^5^ Department of Biology University of Oxford Oxford UK

**Keywords:** cord‐forming, fungal grazers, fungal networks, image‐analysis, modular organisms, morphological traits, phenotypic trajectories, plasticity, soil fauna

## Abstract

Modular organisms such as fungi are assumed to exhibit extreme morphological plasticity, yet this assumption has rarely been tested experimentally. Their morphology emerges from local, independent responses of constituent modules, suggesting strong plastic responses to environmental conditions. While such levels of plasticity decouple morphology from ecological function, they make these organisms an ideal system for studying the evolution of plasticity. Here we quantified the plasticity of modular fungi to grazers with known strong effects on their fitness and tested two competing hypotheses: (1) fungal morphology converges on a common ‘grazing‐resistant’ phenotype across species (i.e., extreme plasticity) or (2) grazer‐induced plasticity remains limited and species‐specific. We found support for the latter, suggesting a more nuanced plasticity for fungi than would be expected based on their modularity. Our study calls for refining assumptions about plasticity in modular organisms and informs the use of morphological traits as predictors of ecological function.

## Graphical abstract

Using image analysis to track the development of fungal networks in the presence of predators, we found that even in modular organisms, plasticity is more nuanced and limited than previously assumed. Our results challenge the long‐held notion that a modular body plan necessarily confers extreme plasticity.

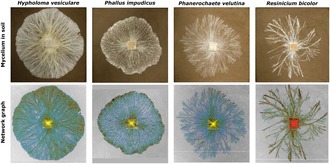

## 1 Introduction

Determining which organisms achieve high levels of phenotypic plasticity has long been a central question in ecology and evolution (Sultan 2003, 2021; West‐Eberhard 1989). In fluctuating environments, plasticity—the ability of a single genotype to alter its phenotype in response to environmental change (Pigliucci and Müller 2010)—is crucial for maintaining or enhancing fitness in the new conditions (Forsman 2015; Pfennig 2021). Although all living organisms show some degree of plasticity, cases of ‘extreme’ or ‘perfect’ plasticity, where individuals can sense environmental change and produce appropriate phenotypic responses throughout their entire lifespan, are rarely observed in nature (Murren et al. 2015). More often, plasticity is ‘constrained’, where phenotypes respond only weakly to the environment, changes may not increase fitness, or the ability to change their phenotype is restricted to narrow windows in their development (Gomulkiewicz and Stinchcombe 2022). This limited occurrence is thought to reflect the inherent costs of plasticity, including the energetic burden of modifying phenotypes and maintaining the regulatory systems needed to sense and respond to environmental variation (Auld et al. 2010; Schneider 2022). However, modular organisms grow by repeating units that can respond independently to local conditions, and it has been suggested that this fundamentally different architecture allows greater plasticity than the fixed body plans of unitary organisms (De Kroon et al. 2005; Murren et al. 2015). Yet, modular lineages remain underrepresented in studies of plasticity, leaving it unclear whether and how often extreme plasticity evolves in such systems.

Filamentous fungi represent a prime group in which to explore the potential for extreme phenotypic plasticity in modular organisms. This group is a dominant lineage in terrestrial ecosystems (Anthony et al. 2023) but has a fundamentally different body plan from that of animals as they grow as networks of interconnected filaments (hyphae) that together form a mycelium (Fricker, Luke, et al. 2017) without a determinate body morphology. Empirical evidence shows that fungi can adjust their morphology in response to environmental variation. In extreme cases, fungi can switch between unicellular (yeast) and filamentous (mycelium) forms depending on conditions such as temperature, pH, or nutrient availability (Boyce and Andrianopoulos 2015; Gauthier 2015). Moreover, the filamentous phenotype allows fungi to alter their morphology to enhance survival under different environmental conditions. For example, in environments where resources are distributed heterogeneously, fungi can alter local space occupancy (e.g., by increasing branching rates) to connect resource patches that are spatially distant or differ in quality, which gives rise to context‐dependent colony patterns (Boddy 1999; Fukasawa and Ishii 2023; Slepecky and Starmer 2009; Veresoglou et al. 2018). Similarly, increases in colony colour, such as melanin deposition in hyphae, provide protection against environmental stressors including UV radiation and desiccation (Dadachova and Casadevall 2008). This morphological flexibility has led to the proposition that fungi exhibit ‘extreme phenotypic plasticity’ (Klein and Paschke 2004; Slepecky and Starmer 2009; Ugalde and Rodriguez‐Urra 2014), and that because of such high levels of plasticity fungi are ‘the most adaptable phylogenetic lineage in the Tree of Life’ (Coleine et al. 2022). Yet, experimental studies quantifying the extent of plasticity in this group are scarce (Alster et al. 2021; Behm and Kiers 2014).

To address this gap, we experimentally assess the extent of plasticity in filamentous fungi to the presence of predators. While the extent of plasticity can be measured in response to any environmental changes, predator‐induced plasticity—where a genotype alters its phenotype in response to the presence of a predator—is particularly informative because it reflects a fundamental component of a species' ecological niche (Morin 2011). Predation is a near universal biotic interaction that imposes strong fitness costs on the prey. As such, it exerts intense evolutionary pressure on organisms to evolve coping strategies, including plastic responses. Indeed, predator‐induced plasticity has been a cornerstone in the development of ecological and evolutionary theory on plasticity (Hoverman and Relyea 2007; Tollrian and Harvell 1999). Predator‐induced morphological plasticity is well documented across diverse animal groups, from classic examples with the model organism Daphnia (Spitze and Sadler 1996) to rotifers (Gilbert 2013; Yin et al. 2017), molluscs (Auld and Relyea 2011), and insects (Flenner et al. 2009; Sentis et al. 2019) among others. Beyond animals, an increasing number of studies show herbivore‐induced morphological changes in plants (Dorey and Schiestl 2022; Fernández De Bobadilla et al. 2022), with responses often depending on the feeding habits of the herbivore (Lebbink et al. 2024).

For filamentous fungi, grazing by soil fauna is analogous to animal predator–prey or plant–herbivore interactions (Boddy and Jones 2007). Grazing severely reduces fungal fitness, particularly during foraging (Tordoff et al. 2006, 2008). For filamentous fungi foraging comes with high fitness costs, as it requires building and maintaining the transport hyphal networks that move resources from nutrient patches to an expanding exploration front in the search of new resources (Bielčik et al. 2019; Tlalka et al. 2002; Wells et al. 1999; Wells and Boddy 1990). Because of these dynamics, even localised damage by grazers could disrupt resource distribution, with potentially long‐lasting and far‐reaching consequences for fungal survival. In response, fungi may adjust their network morphology to maintain function—for instance, by increasing mycelial density (hyphae per unit area), thickening transport routes, or promoting hyphal fusions to create alternative pathways (Rotheray et al. 2008). Such adjustments increase route redundancy, enhancing network robustness—a universal feature of transport systems to preserve function in the face of damage—and thereby enabling fungi to sustain foraging despite grazing pressure (Fricker, Luke, et al. 2017). As grazing is imposed by various different animals, including nematodes, collembola, millipedes and isopods with different types of attack it is likely that plastic responses are specific to distinct grazing behaviour (Crowther et al. 2012).

To determine the extent of grazer‐induced plasticity in filamentous fungi, we reanalyzed an existing image dataset from four fungal species exposed to grazing by a range of soil meso‐ and macro‐invertebrates with different feeding behaviour (Crowther et al. 2011). The experimental design parallels reaction norm studies typically used to evaluate predator‐induced plasticity, where clones of the same genotype were grown with and without grazers. Then we quantified changes in the development of the network architecture in response to grazers using image analysis algorithms (Aguilar‐Trigueros et al. 2022; Fricker et al. 2007; Fricker et al. 2008; Fricker, Luke, et al. 2017; Fricker, Akita, et al. 2017) together with phenotypic trajectory analysis. This method, widely used to study both evolutionary (Adams and Collyer 2009) and developmental plasticity (Metcalfe 2024) using multiple traits (Hoverman and Relyea 2007), has been suggested as a powerful tool for studying fungal plasticity (Behm and Kiers 2014).

**FIGURE 1:**
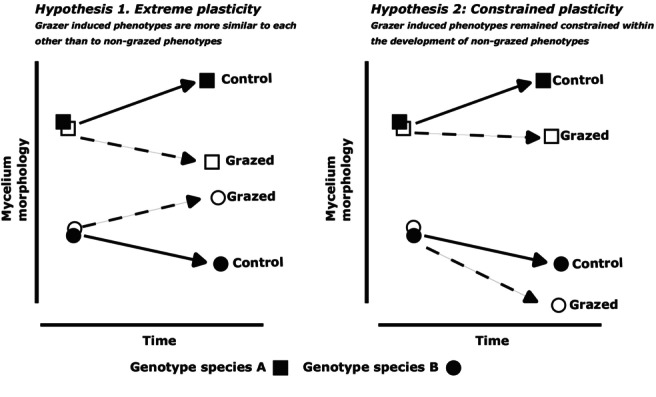
Conceptual representation of hypotheses predicting fungal morphological plasticity in response to grazing. Plasticity in modular organisms like fungi arises through a dual process: Each module senses and responds to environmental change, and these local responses cascade into whole‐body phenotypic shifts, a property maintained throughout vegetative growth. If modularity allows extreme plasticity (Hypothesis 1), grazed individuals will shift their morphology strongly toward grazer‐induced conditions. In this scenario, grazed phenotypes (open symbols, dotted lines) would depart markedly from the non‐grazed phenotypes of the same genotype and instead resemble the grazed phenotypes of other genotypes, converging on grazer‐induced forms. If, despite modularity, plasticity is constrained (Hypothesis 2), morphological changes would still occur yet remain aligned with the trajectory defined by the genotype's inherent morphology rather than converging across genotypes.

Given the high degree of morphological plasticity that the non‐determinate, modular fungal body may confer, together with the strong fitness pressures imposed by grazing, we tested two alternative hypotheses about the extent of plasticity in response to this pressure (Figure 1). Under ‘extreme plasticity’ (Hypothesis 1), the developmental trajectories of fungal phenotypes across fungal species would converge toward a common ‘grazing‐resistant’ phenotype. Alternatively, under constrained plasticity (Hypothesis 2), plastic responses are limited by species‐specific constraints, reflecting evolutionarily more fixed traits despite the modularity of the fungal body. Thus, grazing would result in relatively small morphological shifts in response to grazing, with phenotypes remaining close to species‐specific developmental trajectories. By disentangling these alternatives, our goal was not only to assess the extent of plasticity in filamentous fungi, but also to uncover the extent that modular growth forms mediate adaptive responses to environmental change.

## 2 Materials and Methods

### 2.1 Experimental Setup

The data for this study were derived from a time‐series experiment initially designed to investigate the effects of invertebrate grazing on mycelial foraging (Crowther et al. 2011). The data set includes isolates from four species of wood‐decomposing fungi: *Phallus impudicus*, *Phanerochaete velutina*, *Hypholoma fasiculare*, and *Resinicium bicolor* that were originally isolated from woodlands in the southern UK and are maintained in the Cardiff culture collection (Boddy 1999). During foraging, these four species form hyphal aggregates—termed cords—which can be several mm thick and centimetres long (Boddy 1999; Fricker, Luke, et al. 2017; Fricker, Bebber, and Boddy; Fricker et al. 2008) that can be tracked without a microscope, facilitating image analysis (see below). Differences in morphology arise by changing the rates of cord extension, thickening, branching and fusion of the cords.

Briefly, for each species, 30 clones were grown in microcosms in the lab. Each microcosm consisted of a beech wood block (20 × 20 × 10 mm), previously colonised by the fungi, which served as the sole carbon source. These blocks were placed on compressed woodland soil within lidded trays (25 × 25 cm) and incubated at 18°C. Fungi were allowed to grow and forage from the wood blocks for 8–12 days, until at least half of the mycelial networks reached a diameter of 160–200 mm (see Supporting Information for detailed imaging settings). Five clones per species (i.e., replicates) were then exposed to five grazing treatments, along with a set that was maintained grazer‐free that served as a control: collembola ( *Folsomia candida* ), millipedes ( *Blaniulus guttulatus* ), woodlice sp1 ( *Oniscus asellus* ), woodlice sp2 ( *Porcellio scaber* ), nematode (*Panagrellus redivivus*) and a grazer‐free control.

To monitor mycelial development over time, images were captured immediately before the addition of grazers, and then after 2, 4, 8, 16, and 32 days. Here we restrict analysis up to the 8‐day time point to ensure the network architecture was not perturbed by physical constraints as some species reached the edge of the soil tray dish beyond this day. Images were captured with pixel sizes ranging from 80 to 155 μm.

### 2.2 Quantitation of Mycelial Network Architecture

**FIGURE 2:**
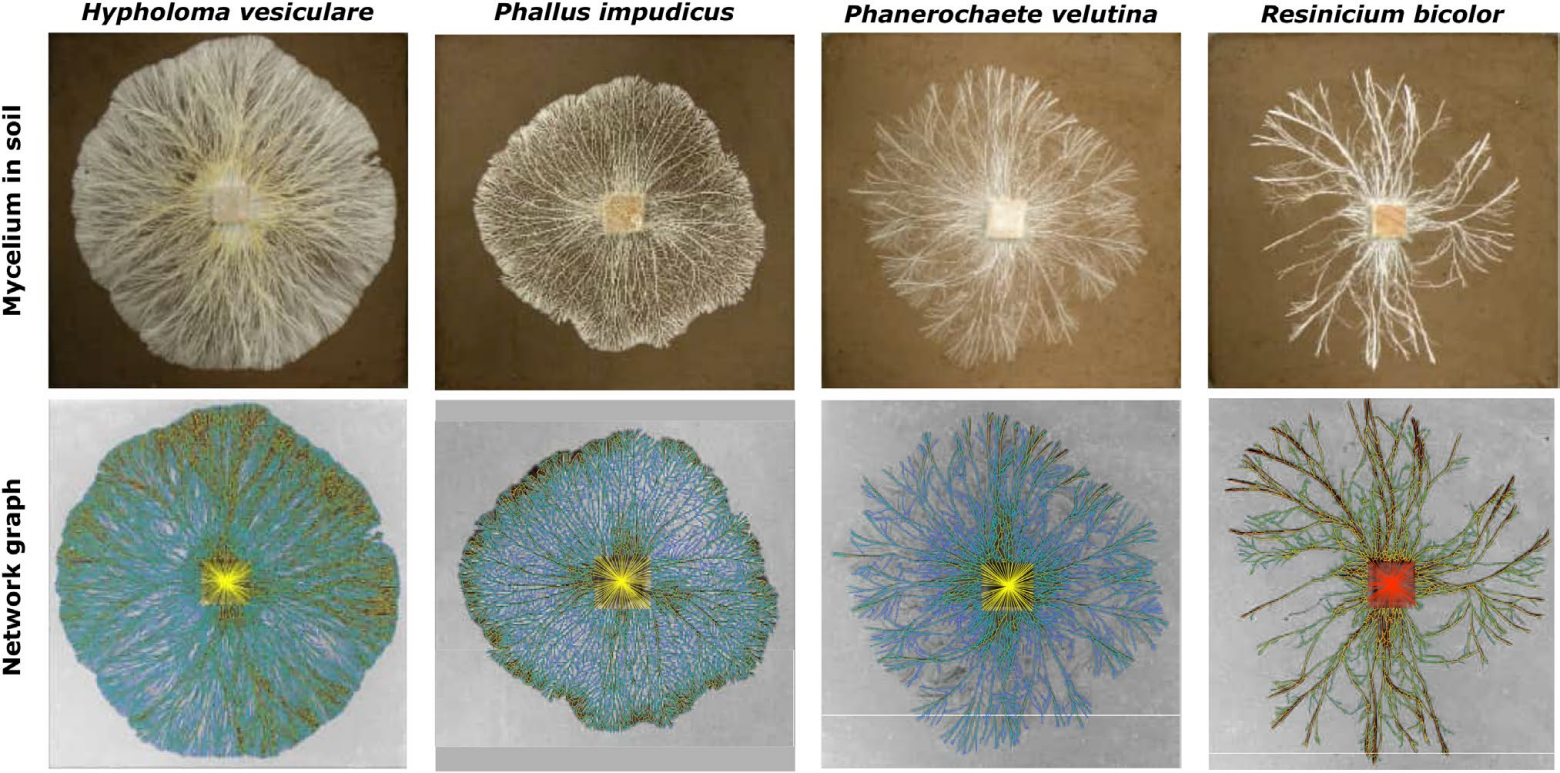
Translating cord‐forming fungi into a weighted transport network. *Top row*: Photographs of mycelia of the four fungal species used in this study. Mycelia have grown from 2 × 2 × 1 cm beech wood blocks, over the surface of compressed woodland soil (water potential −0.012 MPa) in 24 × 24 cm trays, incubated at 21°C. *Bottom row*: Network graphs overlaid on black‐and‐white images of the mycelia, generated using an image‐processing algorithm that skeletonized the cords and measured cord width. Cords are represented as network edges (also called links); and tips, branching points, and fusion points (anastomoses) are represented as nodes (also called vertices). Cord width is visualised with colour: Green for thick cords, blue for thin cords. For visualisation, the inoculum is represented as multiple linear edges in yellow, but these were not included in the analysis.

We used a set of image processing algorithms to extract the mycelial morphology/architecture from photographs as described in Aguilar‐Trigueros et al. (2022). Briefly, each picture was loaded into a graphical user interface (GUI, Figure S1) and processed through a standard pipeline that included background correction, network enhancement, segmentation, skeletonization, width estimation and network graph representation using protocols optimised across a range of biological network systems (Fricker, Luke, et al. 2017; Fricker, Akita, et al. 2017; Obara et al. 2012; Pain et al. 2019; Xu et al. 2021). The output of this pipeline is a network graph representation of the mycelia where the tips, cord branching and cord fusion events are nodes whilst the cords themselves form ‘edges’ connecting the nodes (Figure S1). The length and width of each edge were automatically measured, whilst the (*x*, *y*) position and branching angle were recorded for each node. It was not possible to resolve edges within the inoculum, so these are represented as strong connections between each incident edge on the resource and a central node (termed the ‘Root’ of the network). This information was exported in two tables as .csv and .xlsx files containing the cord (edge) information and the tips, branching and fusion points (node) information. The node and edge tables from the image processing pipeline were imported into R (using the *read_excel* function from the readxl package; Wickham et al. 2019) and used to construct a spatially explicit transport network embedded in 2D space given by the Euclidean coordinates of the nodes using the igraph package in R (Csardi and Nepusz 2006) (Figure 2).

### 2.3 Traits Used to Define Mycelial Network Morphology

Network morphology was measured using a set of 21 network traits that reflect the overall colony morphology, network heterogeneity, space filling and connectivity, predicted transport and predicted robustness to damage of the network (Table S1). We adopted this multivariate trait approach because mycelium morphology is a complex phenotype that cannot be captured by a single trait (such as body size or aspect ratios used in animals). To isolate the plastic response of network traits to grazers—rather than the effects of grazers directly consuming the mycelium—we implemented two approaches. First, we normalised trait values relative to those of the network's *Minimum Spanning Tree* (MST). In our mycelial networks, the MST represents a tree‐like subset with only one possible path connecting any two nodes. It consists of the cords with the lowest predicted transport resistance between the wood block (inoculum) and the foraging tips at the colony front and thus represents the primary transport routes of the network. Since predicted transport resistance scales to the inverse of cord width (Bebber et al. 2007; Fricker et al. 2007), the thickest cords of the network are prioritised in the identification of the MST. Cords not part of the MST were classified as secondary routes that contribute to the cross‐linking of the network. By normalising to the MST, we focused on relative deviations in morphology, rather than absolute values—capturing whether grazers shifted the network toward or away from the MST morphology. Second, we restricted trait measurements to the portion of the mycelium that is attached to the wood block. This approach ensured that only the mycelium that has access to resources that assist foraging and morphological development was analysed, excluding any mycelial fragments that had been detached due to direct consumption of the grazer.

### 2.4 Phenotypic Trajectory Analysis to Measure Morphological Developmental Plasticity

Phenotypic trajectory analysis relies on the use of multivariate ordination methods to summarise the change through time in phenotypes across multiple traits combined using ANOVA‐like frameworks for statistical testing (Adams and Collyer 2009; Behm and Kiers 2014). In this study, we used three types of permutational multivariate analyses of variance models (PERMANOVA) to determine the level of grazer‐induced plasticity across the fungal species using the 22 metrics. In all the models, we controlled for initial differences in inoculum growth at the start of the experiment by using the first two axes from a principal component analysis (PCA) of traits at time 1 (i.e., immediately before the addition of the grazers) as a covariate in the PERMANOVA.

In the first and more general model, we tested whether induced changes in the development of the morphology (plasticity) converge to a common grazing‐resistant phenotype across all four species (Hypothesis 1, extreme plasticity) or whether the plastic response remained constrained close to the trajectories of the non‐grazed phenotype per species. In this model, time, fungal species identity, and grazer type were used as fixed factors and PC1 and PC2 of the traits were used at the start of the experiment to control for initial conditions (see Table 1 for the full formula used in this model). If fungal species converge to a common phenotype, we expect to detect a strong (and significant) main effect of grazer together with an interaction between grazer and time. That is, a strong interaction grazer × time suggests more deviation in the development of the morphology of grazer‐exposed clones compared to the grazer‐free clones, while a strong main grazer effect suggests similarity in the morphology of the grazed phenotype.

**TABLE 1:** PERMANOVA results with effect sizes for the first PERMANOVA model that included fungal species, grazer identity and developmental time.

Source	df	Variance	*F*	*R* ^2^	Pr(> *F*)
Species	3	8.5397	208.4704	0.4314	< 0.001
Grazer	5	0.5294	7.7541	0.0267	< 0.001
Day	1	0.8777	64.2807	0.0443	< 0.001
Species:day	3	1.1389	27.8028	0.0575	< 0.001
Species:grazer	15	1.2921	6.3084	0.0653	< 0.001
Grazer:day	5	0.1542	2.2581	0.0078	< 0.001
Species:grazer:day	15	0.5309	2.5923	0.0268	< 0.001
Residual	493	6.7317			

*Note:* The variation in the initial condition for each colony was corrected using PC1 and PC2 coordinates for day 1 as covariates.

In the second model, we determined which grazers induced a common phenotype at the end of the experiment. We ran five PERMANOVA models (i.e., one for each grazer) where the morphological traits of all four fungal species were used as response variables while the only fixed factor was the grazer treatment with two levels (presence or absence). Convergence would be shown by a significant, large effect size of grazer identity. To further visualise and validate the output of these models, we tested whether the grazers increased the similarities among fungal species compared to the control. This test was done by measuring the distances between each fungal species, across the respective grazer treatments, to their multivariate mean (centroids) followed by a permutation‐based test for multivariate homogeneity of group dispersions (implemented with the function *betadisper* from the package *vegan* in R). If grazers change the similarities of the morphological phenotypes of the four genotypes it would be shown as a strong and significant reduction in the distances between each species and their respective centroids.

The third model tested which fungal species had the most plastic response to grazers. To do so, we ran PERMANOVA models for each fungal species. Thus, this test measured the extent of developmental plasticity of fungal species irrespective of convergence to a common phenotype. Finally, for the fungal species showing the largest plastic responses, we performed a last series of models with each of their grazers separately to further identify which trait drove the plastic responses.

In all PERMANOVA models, we used a forward model selection approach to determine which fixed factors should be retained. We used principal component analysis (PCA) to visualise the changes in the phenotype through time. Analysis of the distances to each centroid was done using Euclidean distances. Effect sizes were measured as the proportion of explained variance of the fixed factors. PERMANOVA, PCA, analysis of distances to centroids, model selection and effect sizes were determined using functions from the vegan package (Oksanen et al. 2007). All statistical analyses were conducted in R (R Core Team 2013). Code and data used in this study are found in GitHub and Zenodo repositories: https://github.com/aguilart/FungalGrazers and https://doi.org/10.5281/zenodo.1493195.

## 3 Results

### 3.1 Fungal Morphology Showed Limited Plasticity to Fungal Grazers

**FIGURE 3:**
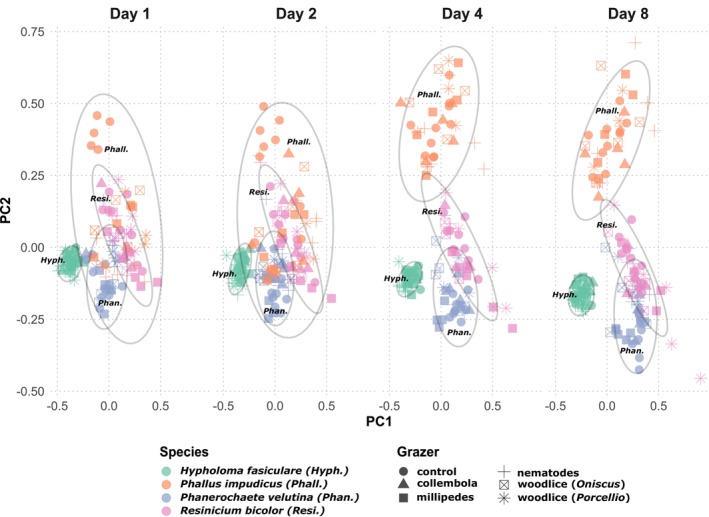
Principal component analysis (PCA) showing the temporal development of mycelial morphology in four fungal species. Over time, species‐ and genotype‐specific morphological trajectories became increasingly distinct. Although the presence of grazers altered these trajectories, the resulting phenotypes remained constrained within the morphological space characteristic of each genotype (indicated by ellipses).

Species identity had the strongest effect, explaining up to 90% of the variation in morphology across all treatments and time points using model 1 (Figure 3). There were significant effects of grazer identity and an interaction with time, but neither effect was larger than 5% of the total variation (Table 1).

In the second set of models, whose focus was on the effect of each grazer separately, there was no support for convergence to a common phenotype; instead there was a significant effect for increasing dispersion among genotypes in grazer‐induced morphology (except with collembola for which the effect was not significant) (Figure S2). Effect size varied from 5% to 10% and was strongest for woodlice sp1 ( *O. asellus* ), followed by millipede, woodlice sp2 ( *P. scaber* ) and nematode treatment. Taken together, these results show that grazer‐induced plasticity was detectable across fungal species, but it was constrained within the trajectory of each genotype and that instead of converging to a common phenotype it increased the dissimilarity of the phenotypes.

### 3.2 Trajectories of Grazer‐Induced Plasticity Differed in Magnitude and Direction Among Fungal Species

**FIGURE 4:**
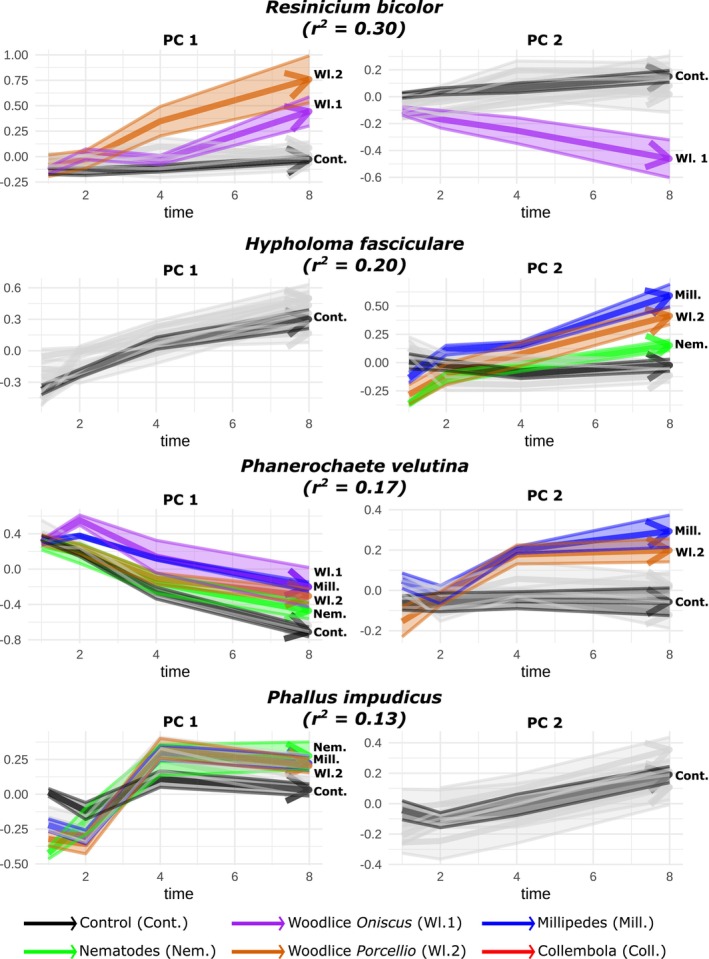
Species‐specific trajectories of mycelial morphological development over time and deviations caused by grazer presence. The greatest grazer‐induced deviation was in *R. bicolor* , where grazers explained 30% of the variation in morphology through time, followed by *H. fasciculare*, *P. velutina* , and *P. impudicus* . Trajectories of grazer‐induced morphologies that differ by more than twice the joint standard error from the non‐grazed phenotypes are colour‐coded to highlight these contrasts.

**FIGURE 5:**
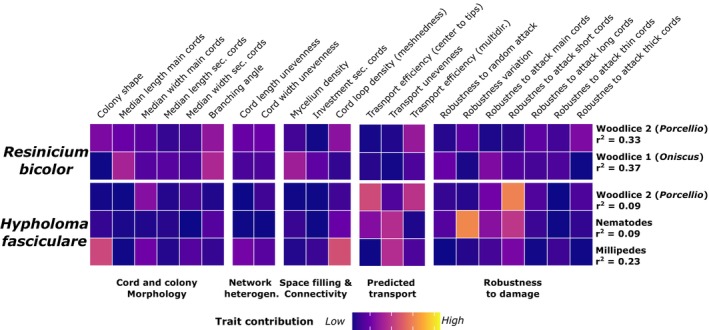
Heatmap of morphological trait shifts between grazed and non‐grazed genotypes at the end of the experiment for *R. bicolor* and *H. fasciculare*. These two species showed the strongest grazer‐induced deviations in morphology (see Figure 4). For each species, the grazer treatments with the largest deviation are shown, along with the percentage of explained variation. Corresponding RDA biplots are in Figure S3.

There was large variation in the level of plasticity among the fungal species tested across the grazers (Figure 4). *R. bicolor* exhibited the highest morphological plasticity in response to grazers, with up to 30% of the variation in morphology altered in response to grazing (*p* < 0.01), particularly in response to the two woodlice species (Figure 4). In both cases, RDA plots revealed clear separation between grazed and ungrazed genotypes of this species (Figure S3). Multiple traits contributed to this divergence—indicating a broadly distributed response rather than dominance by a single or few traits (Figure 5). Among the two woodlice treatments, woodlouse 1 (*Oniscus*) had the stronger effect. While overall colony shape did not change when exposed to this grazer, there was a change in cord morphology shifting to shorter, thinner cords with straighter branching angles. A more prominent change was the increase in cord density, although loop density was unaffected. Despite these structural changes, the predicted transport dynamics remained stable, and robustness even improved in this treatment, particularly against random attacks or targeted attacks on main cords.

Colonies exposed to woodlice sp2 (*Porcellio*) became more circular and exhibited similar changes in cord morphology—that is, they had shorter and thinner cords with straight branching angles. Cord heterogeneity also shifted: cord lengths became more homogenous (less skewed distribution compared to non‐grazed ones), while width distribution became more uneven, suggesting the emergence of a few dominant thick cords. In this treatment, the number of loops decreased while density remained stable. These changes were associated with reduced predicted transport efficiency, especially for multidirectional transport, and increased variability in robustness to random attacks. Importantly, robustness to attacks on thick cords declined, indicating these few thick cords were critical for network connectivity and vulnerable to disruption.

*H. fasiculare* showed the second‐highest response with ca. 20% (*p* < 0.05) of morphological variation explained by the presence of the grazer (Figure 4). This effect was primarily driven by exposure to millipedes with up to 23% explained variation attributed to this grazer. In this case, only a subset of traits shifted, including the development of a more irregular colony shape and a marked decrease in loop number. These structural changes were associated with a reduction in predicted transport efficiency and a sharp increase in the heterogeneity of main transport cords. However, predicted robustness remained largely unaffected, with only a slight increase in robustness to attacks on thin cords. Responses to woodlice sp2 (*Porcellio*) and nematodes were weaker (with around 9% of explained variation attributed to the presence of these grazers; Figure 5) and more variable across replicates, as reflected in less consistent RDA separation (Figure S3).

*P. velutina* and *P. impudicus* exhibited the lowest morphological plasticity, with only 17% and 13% of trait variation attributed to grazer effects, respectively (Figure 4). Although these effects were statistically significant, RDA plots showed inconsistent separation between treatment groups (Figure S3), suggesting limited and variable responses across replicates.

## 4 Discussion

We tested whether grazing pressure would induce convergence toward a common mycelial phenotype, or alternatively, whether plastic responses would remain constrained within the morphological trajectories of non‐grazed genotypes. Our results support the latter: although grazing altered mycelial morphology in some species, these effects were modest, and genotypes consistently retained their characteristic morphology across all treatments. Moreover, the morphological changes induced by the grazers led to increased dissimilarities among grazed genotypes rather than convergence to a particular grazing‐resistant phenotype. These results suggest that the mycelial morphology has more limited flexibility to reconfigure itself and that morphological plasticity likely plays a minor role in dealing with grazing pressure.

### 4.1 Strong Species Differences Suggest Limits in Morphological Plasticity

Although some species showed notable grazer‐induced plasticity, genotype‐specific differences still dominated. Plasticity thus appears bounded within species‐specific limits. These limits also differed among species: *R. bicolor* changed by ~30%, whereas *P. impudicus* shifted only ~10%. Low plasticity in *P. impudicus* may reflect constitutive, grazing‐resistant architecture (e.g., dense cross‐linking), or higher metabolic costs of remodelling its mycelium relative to investing in other defences (Auld et al. 2010). Metabolic costs of modifying the morphology of the network may be related to differences in the anatomy and histology of the cords among species (Thompson and Rayner 1983; Yafetto 2018; Townsend 1954). *P. impudicus* has greater differentiation of anatomical regions within the cord compared to that of other cord‐forming fungi (Eamus et al. 1985; Townsend 1954). *P. velutina* , also showing relatively low plasticity in this study, has cords that are differentiated internally (Thompson and Rayner 1983). In contrast *H. fasciculare*, with relatively higher levels of plasticity in our study, is reported to be on the lower end of the cord complexity spectrum (Thompson and Rayner 1983). There are few anatomical studies of the cords of *R. bicolor* (the species with the most plasticity in our study), but it is a fast‐growing species (Zakaria and Boddy 2002) a life‐history trait that is commonly associated with lower investment in resources in body construction in other organisms (Wright et al. 2004). Thus, a trade‐off may exist between the ability to respond to grazer pressure and increasing complexity of cords. Future studies comparing the anatomy of the cords across species are needed to test this hypothesis.

Weak plastic responses, like those in our study, may indicate that organisms rely on rapid evolutionary adaptation to cope with environmental change (Vinton et al. 2022). Rapid evolution has been documented in some fungi, for instance in plant pathogens adapting to host immune systems (Kusch et al. 2024) and in sex‐related genes of the model species *Neurospora crassa* (Nygren et al. 2012), both characterised by short generation times, fast growth, and large populations. There have been no studies testing the role of rapid evolution in cord‐forming fungi; however, some of the life‐history traits of cord‐forming fungi, such as annual sexual cycles and long‐lived and large clonal phases (Anderson et al. 2018; Kauserud et al. 2011; Thompson and Rayner 1983), suggest restricted potential for rapid evolution.

### 4.2 Low Plasticity and Lack of Convergent Responses Suggest Weak Evolutionary Pressure of Fungal Grazing for Induced Morphological Plasticity

Our results indicate that soil grazers do not trigger a strong plastic response in mycelial phenotypes. Even in the most responsive species (e.g., *R. bicolor* ), the true extent of plasticity may be lower as the observed phenotypic variation may still be influenced by direct grazing damage—despite our efforts to normalise for this confounding effect. In addition, although we used an 8‐day cut‐off for recording changes in mycelia due to size constraints, our results are congruent with the small differences in cord density documented between grazed and non‐grazed individuals of *P. velutina* after 208 days in larger 0.5 × 0.5 m trays of soil (Boddy et al. 2010; Wood et al. 2006). Together, these results suggest that morphological plasticity plays a limited role in how fungi respond to grazing and do not support the idea that grazing exerts strong evolutionary pressure for adaptive morphological traits. It could be that optimising morphology for one grazer may reduce adaptability to others and, even if an optimal phenotype exists, fungi may spend extended periods in suboptimal, transitional forms—indicative of weak or diffuse selection (Einum and Burton 2023). Grazing, however, could still induce plasticity in non‐morphological traits, such as physiological or biochemical changes—so‐called ‘hidden plasticity’ (Forsman 2015), such as chemical defences that reduce palatability or increase toxicity to grazers (Connolly et al. 1999). This is a strategy well documented in plants (Fernández De Bobadilla et al. 2022) which could occur in fungi (Lee et al. 2020; Plaza et al. 2016; Schmieder et al. 2019).

### 4.3 Implications of Plasticity Limits in Trait‐Based Fungal Ecology

The pronounced interspecific differences but limited plasticity in mycelium morphology suggest that morphological traits can serve as functional traits for linking community composition to ecosystem processes (Mcgill et al. 2006). When a trait changes drastically across environments without affecting species function, it becomes an unreliable proxy for ecological role. Traits with lower plasticity are therefore preferred for inferring and predicting species distributions and ecosystem functioning along environmental gradients (Franklin et al. 2020; HilleRisLambers et al. 2012). In fungi, mycelial traits may thus help predict how shifts in community composition under environmental change affect ecosystem processes such as carbon cycling (Dawson et al. 2024). Cord‐forming fungi, for instance, play a key role in ligno‐cellulose decomposition, and morphological traits may influence decomposition efficiency, thereby affecting carbon budgets.

Grazer‐induced plasticity, though, may not reflect the full potential for morphological responses to other environmental factors. For example, considerable variation among replicates of the same genotype was present at the experiment's start (Figure S4), likely due to differences in early growth, resource quality, or wood block colonisation. Thus, while plasticity to grazers appears limited, other cues such as resource heterogeneity may trigger stronger morphological responses. These findings warrant further exploration of the predictable relationships between mycelial morphology, environmental conditions, and ecosystem functions.

### 4.4 Implications of Using Fungi as Model Systems for Studying the Ecology and Evolution of Plasticity

This study highlights cord‐forming fungi as a tractable and underutilised model for investigating plastic responses to grazing in modular organisms. Their rapid growth and compatibility with laboratory conditions make them well suited for exploring the extent, nature and mechanisms of morphological plasticity. Future experiments using split‐compartment designs—where grazers interact with only part of the mycelium while traits are measured in grazer‐free zones—could help determine whether plasticity is systemic across the network, even in ungrazed areas. Such designs, which have previously been used in these types of soil microcosms (A'Bear et al. 2013), would also allow clearer separation of plastic responses from direct consumption effects which could not be completely disentangled in our study. Likewise, legacy‐type experiments, in which phenotypes are assessed after grazer removal or in a subsequent generation of the clone (e.g., Fukasawa et al. 2020), could help reveal the persistence and underlying mechanisms of the response, including potential epigenetic effects. Together, these complementary approaches would extend our framework and advance understanding of plasticity in modular systems.

Moving beyond cord‐forming fungi, it remains to be tested whether fungi that did not evolve the capacity to aggregate hyphae exhibit the expected extreme plasticity attributed to fungi (Klein and Paschke 2004; Slepecky and Starmer 2009). Given that phenotypic plasticity tends to evolve in highly variable environments (Lande 2009), fungi inhabiting soils—among the most heterogeneous habitats—would be expected to show strong selective pressures for high morphological plasticity. Evolutionary models suggest that filamentous growth forms, which are particularly advantageous in spatially heterogeneous environments like soils, would not have evolved in more homogeneous aquatic habitats (Heaton et al. 2020). This parallels findings in ectotherms, where greater plasticity is observed in terrestrial species compared to aquatic ones, likely due to higher environmental variability on land (Einum and Burton 2023). However, our results suggest limited plasticity in cord‐forming fungi, a phenotype that has evolved repeatedly in the Basidiomycota (Monk 2014). Such a complex and energetically costly phenotype may have evolved to optimise efficiency in specific ecological roles. This specialisation could limit the need for extensive morphological plasticity.

Our study underscores the importance of understanding plasticity in modular organisms. For fungi, our results suggest that, despite the flexibility inherent to this body type, mycelial morphology may be more constrained across species than often assumed. Confirming this pattern across a broader range of species and environments would establish mycelial morphology as a functional trait for predicting species responses to environmental change—providing a much‐needed tool for the emerging field of trait‐based fungal ecology. On a more fundamental level, fungi offer an ideal system to investigate the mechanics of modularity itself. Plasticity in modular organisms operates at two distinct levels—the individual module and the collective organism—raising critical questions: How are these levels coordinated to produce an effective environmental response, and how does selection act upon each? Given that modularity is a convergent trait across the Tree of Life, addressing these questions in fungi promises foundational insights into the evolution of all modular organisms.

## Author Contributions

C.A.A.‐T. and M.D.F. conceived the paper. L.B. supervised the original experiment and provided images of the mycelial systems, metadata and ecological insight. M.D.F. performed the image analysis; C.A.A.‐T. did the statistical analysis. C.A.A.‐T. wrote the first version of the manuscript. All coauthors contributed to writing the final version of the manuscript.

## Conflicts of Interest

The authors declare no conflicts of interest.

## Supporting information


**Data S1:** ele70281‐sup‐0001‐DataS1.docx.

## Data Availability

All data and code used in this study is openly available in a GitHub and Zenodo repository: https://github.com/aguilart/FungalGrazers and https://doi.org/10.5281/zenodo.14931953.
