# Supplementary material for: Not Extremely Plastic: Testing the Limits of Morphological Plasticity in Fungal Mycelia in Response to Soil Grazers
Source: Ecol Lett. 2025 Dec 3;28(12):e70281. doi: 10.1111/ele.70281 (PMC12675975; doi:10.1111/ele.70281)
Supplement: Supplementary file 1 — Data S1: ele70281‐sup‐0001‐DataS1.docx. [file ELE-28-0-s001.docx]

**Supplementary material**

**Title:** **Not Extremely Plastic: Testing the Limits of Morphological Plasticity in Fungal Mycelia in Response to Soil Grazers**

**Supplementary methods**

**Supplementary Table S1**

**Supplementary Figure S1-S3**

**Supplementary Methods**

**Image processing**

Multiple image processing algorithm have been used to segment images of fungal mycelia so data on network properties can be extracted (Dikec *et al.* 2020; Du *et al.* 2016; Fricker *et al.* 2017; Heaton *et al.* 2012; Sten *et al.* 2024; Vidal-Diez De Ulzurrun *et al.* 2015). The choice of algorithms depends on the contrast between the specimen to the background and the light method used among others. For the images of macroscopic mycelia analysed in this study with cords of different thickness at milimeters scale and use of reflective light, the mean-phase angle from intensity-independent phase congruency analysis gives marginally better network enhancement performance compared to other standard approaches such as second-order anisotropic Gaussian (Lopez-Molina *et al.* 2015; Vidal-Diez De Ulzurrun *et al.* 2015) or Bowler-Hat (Oyarte Galvez *et al.* 2025) algorithms used for networks grown on transparent media and with transmission light. Our software, however, has the functionality to implement any of the algorithms used. The software package and complete manual, explaining in detail the steps involved, is freely available from Zenodo (DOI: [10.5281/zenodo.5187932](https://doi.org/10.5281/zenodo.5187932)).

**References**

Dikec, J., Olivier, A., Bobée, C., D’Angelo, Y., Catellier, R., David, P., *et al.* (2020). Hyphal network whole field imaging allows for accurate estimation of anastomosis rates and branching dynamics of the filamentous fungus Podospora anserina. *Sci Rep*, 10, 3131.

Du, H., Lv, P., Ayouz, M., Besserer, A. & Perré, P. (2016). Morphological Characterization and Quantification of the Mycelial Growth of the Brown-Rot Fungus Postia placenta for Modeling Purposes. *PLOS ONE*, 11, e0162469.

Fricker, M.D., Akita, Heaton, L.L.M., Jones, N., Obara, B. & Nakagaki, T. (2017). Automated analysis of Physarum network structure and dynamics. *Journal of Physics D: Applied Physics*, 50, 254005.

Heaton, L.L.M., Obara, B., Grau, V., Jones, N., Nakagaki, T., Boddy, L., *et al.* (2012). Analysis of fungal networks. *Fungal Biology Reviews*, 26, 12–29.

Lopez-Molina, C., Vidal-Diez De Ulzurrun, G., Baetens, J.M., Van Den Bulcke, J. & De Baets, B. (2015). Unsupervised ridge detection using second order anisotropic Gaussian kernels. *Signal Processing*, 116, 55–67.

Oyarte Galvez, L., Bisot, C., Bourrianne, P., Cargill, R., Klein, M., van Son, M., *et al.* (2025). A travelling-wave strategy for plant–fungal trade. *Nature*.

Sten, O., Del Dottore, E., Pugno, N. & Mazzolai, B. (2024). A ridge-based detection algorithm with filament overlap identification for 2D mycelium network analysis. *Ecological Informatics*, 82, 102670.

Vidal-Diez De Ulzurrun, G., Baetens, J.M., Van Den Bulcke, J., Lopez-Molina, C., De Windt, I. & De Baets, B. (2015). Automated image-based analysis of spatio-temporal fungal dynamics. *Fungal Genetics and Biology*, 84, 12–25.

**Table S1. Extended description of the five trait types used in this study**. These traits summarize different patterns of the whole mycelium and are measured through multiple metrics reflecting the construction of the network, density, average and variation in expected transport and robustness to change.

| **Type** | **Biological meaning** | **Trait** | **Metric** |
| --- | --- | --- | --- |
| 1. **Cord and colony morphology** | Size and shape of the whole colony and of the cords | Median cord length and width (main and secondary routes), median branching angle, colony circularity. | (1) Colony circularity, (2) median length of main cords, (3) median length of secondary cords, (4) median width of main cords; (5) median width of secondary cords; (6) branching angle. |
| 1. **Network heterogeneity** | Variation in how fungi build cords within the network | Skewness of cord width and length of main routes. | (7) Skewness length of main cords; (8) skewness width of main cords. |
| 1. **Space filling and connectivity** | Area explored for resources and extent of network interconnection | Mycelial density, loop density (meshnedness), investment in secondary routes. | (9) Cord length per unit of area; (10) alpha coefficient*; (11) ratio total mycelial volume to mycelial volume main cords. |
| 1. **Predicted transport**** | Estimated average transport flow through cords and its variation within the network | Expected average transport from inoculum to margin, expected multidirectional transport, skewness of transport from inoculum to margin. | (12) expected transport efficiency to mycelium front; (13) expected transport efficiency through the network; (14) skewness of expected transport efficiency of main cords |
| 1. **Predicted robustness to damage***** | Expected robustness to removal of cords of the network | Percentage of cords that can be removed before network volume drops below 50%; removal scenarios include random, targeted by cord width, and targeted by cord length. | (15) robustness to attack of main cords; (16) robustness to random attack of the network; (17) variation in robustness to random attack, robustness to attack of thin (18), thick (19), short (20), and long (21) cords. |

* Here, the alpha coefficient (also referred to as *meshedness*) quantifies the number of loops formed by cords relative to the overall structure of the fungal network. In this context, a *loop* is defined as a closed structure formed by at least three interconnected cords. The alpha coefficient is a metric derived from network science that estimates the density of such loops, calculated using the formula:
(ecount(x) – vcount(x) + 1) / (2 × vcount(x) – 5), where *ecount(x)* is the number of cords (edges) and *vcount(x)* is the number of nodes (branching and tip points) in the network.

** Predicted transport in the network was simplified as unidirectional fluid flow from the wood block to the growing front of the network by assuming cords can be represented as a contiguous network of interconnected bundles of cylindrical shape. Predicted transport scales with to the square of the radius of the cord and inversely to cord length (i.e. the inverse of flow *resistance µ l/r^2^*) (Bebber *et al.* 2007; Fricker *et al.* 2007a). This is a simplification of transport dynamics with mycelia but matches well with empirical distributions of radiolabeled nutrient movement in these networks (Heaton *et al.* 2010, 2012a).

******* Predicted transport was assessed *in silico* by simulating the removal of cords from the network. Mycelial volume (biovolume) connected to the inoculum was calculated as the sum of all cord segment volumes still connected after damage, assuming cords are perfect cylinders.


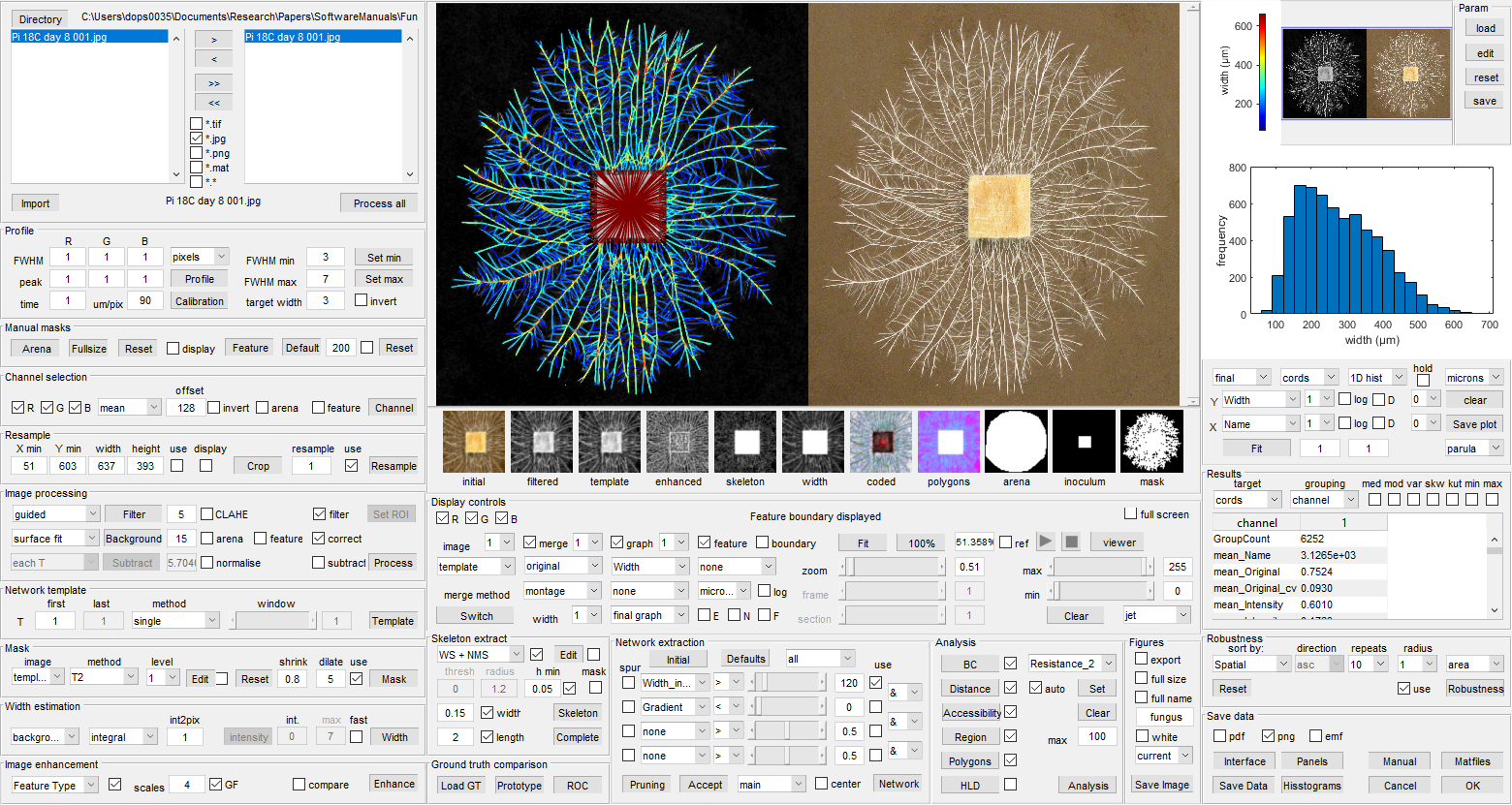


Figure S1. GUI

**
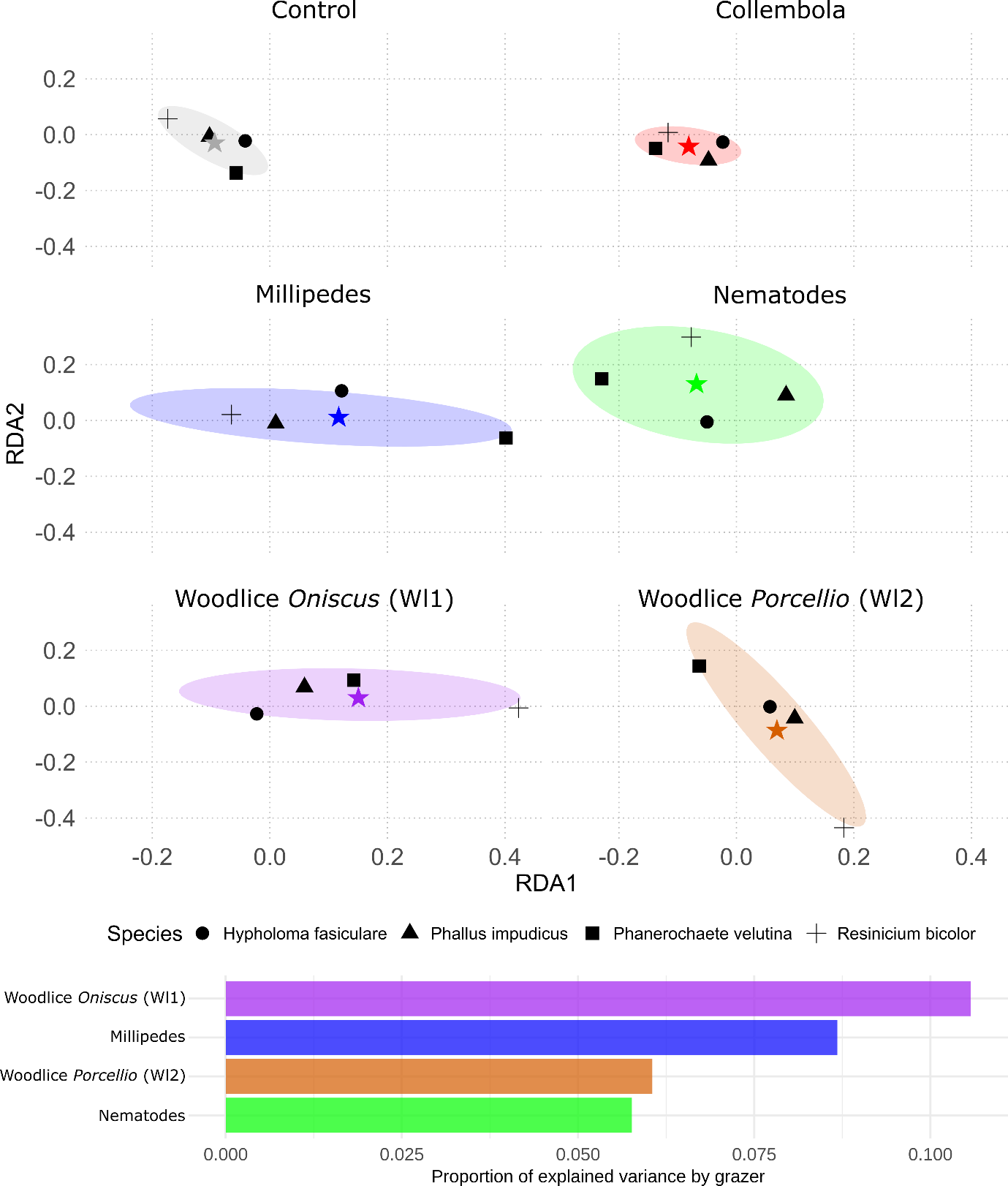
**

**Figure S2. Testing for convergence or divergence in fungal phenotypes under grazer pressure.** Each ordination plot shows the average mycelial morphotype of each genotype for each grazing treatment:circle = *H. fasciculare*, triangle = *P. impudicus*, square = *P. velutina*, cross = *R. bicolor*). The across-genotype average for each grazer treatment is shown as a star. Dispersion around this centroid is visualized with shaded ellipses. With the exception of collembola, where no significant effect was detected, all grazer treatments increased morphological dispersion. Bar plots at the bottom indicate the effect size of grazer-induced changes in dispersion for significant treatments.


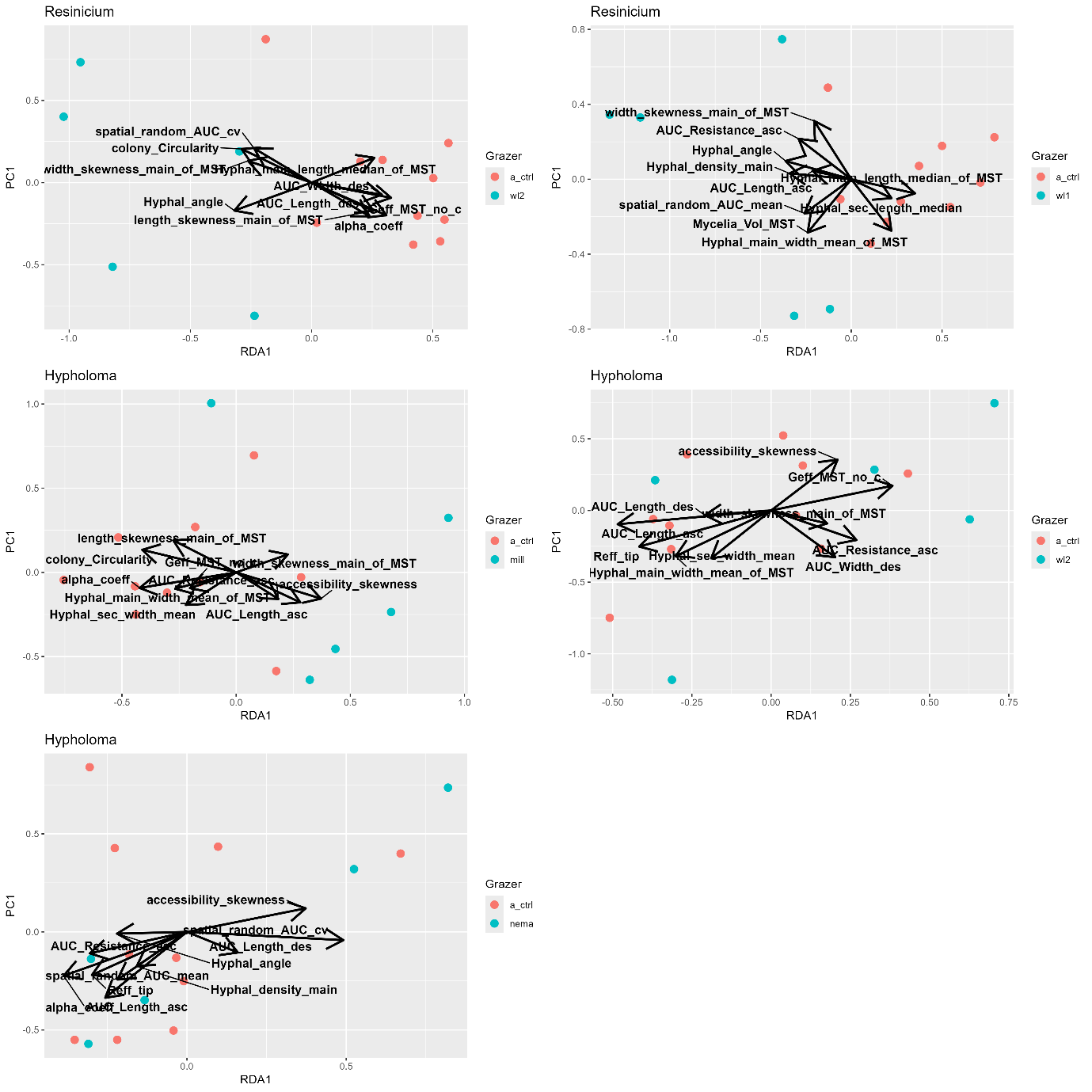


Figure S3. RDA plots showing separation of fungi with highest effect sizes.


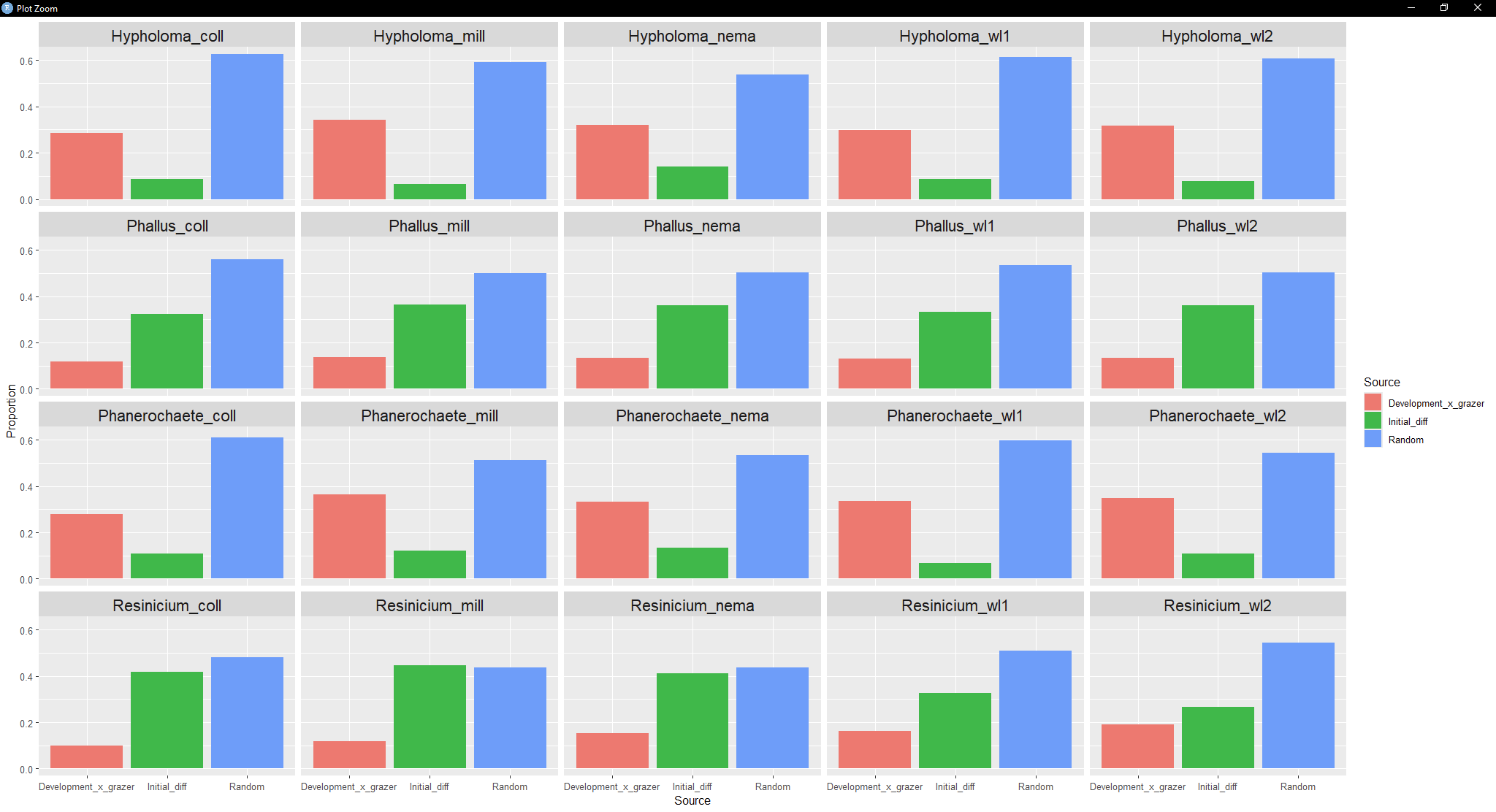


Fig S4. Variance partition among variables in the RDA. Each bar plot represents the proportion of explained variation by the explanatory variables in the RDA (that is, by the time and presence/absence of grazers), the initial growth before the addition of grazer and unaccounted (random) variation.
